# Fit to Serve? Exploring Mental and Physical Health and Well-Being Among Transgender Active-Duty Service Members and Veterans in the U.S. Military

**DOI:** 10.1089/trgh.2015.0002

**Published:** 2016-01-01

**Authors:** Brandon J. Hill, Alida Bouris, Joshua Trey Barnett, Dayna Walker

**Affiliations:** ^1^ Center for Interdisciplinary Inquiry and Innovation in Sexual and Reproductive Health (Ci3), Department of Obstetrics and Gynecology, The University of Chicago, Chicago, Illinois.; ^2^ Section of Family Planning and Contraceptive Research, Department of Obstetrics and Gynecology, The University of Chicago, Chicago, Illinois.; ^3^ School of Social Service Administration (SSA), The University of Chicago, Chicago, Illinois.; ^4^ Department of Communication, University of Utah, Salt Lake City, Utah.; ^5^ Transgender American Veterans Association (TAVA), Tampa, Florida.

**Keywords:** mental health, military/Department of Defense, physical health, transgender, transgender health

## Abstract

**Purpose:** Although transgender people are currently excluded from enlistment and discharged from service based on medical and psychological fitness policies, the current mental and physical health of transgender active-duty U.S. military personnel and veterans is poorly understood. The purpose of the current study was to investigate the military histories, lifetime mental and physical health diagnoses, and transgender transition-related health of transgender active-duty service members (ADSM) and veterans.

**Methods:** Participants were recruited through private LGBT military and veteran organizational listservs, snowball sampling, and in-person recruitment to complete an anonymous and confidential self-administered online questionnaire.

**Results:** A total of 106 transgender ADSM (*n*=55) and veterans (*n*=51) completed the questionnaire. Transgender veterans were significantly older (44 mean years vs. 29.5 mean years, *t*=−6.23, *p*<0.001). A greater percentage of veterans than ADSM reported depression (64.6% vs. 30.9%, *χ*^2^=11.68, *p*=0.001) and anxiety (41.3% vs. 18.2%, *χ*^2^=6.54, *p*=0.011). In addition, 15.9% of veterans versus 1.8% of ADSM (*χ*^2^=6.53, *p*=0.011) had been diagnosed with a substance abuse disorder. There were no significant differences in lifetime physical health conditions; however, veterans reported a higher body–mass index than ADSM (28.4 vs. 24.9, *t*=−3.85, *p*<0.001). For both groups, mental and physical health problems were positively correlated with age and years of military service (*r*=0.37–0.84, *p*<0.01). There were no significant differences between groups in transgender transition-related health.

**Conclusion:** Our data represent the first descriptive statistics of lifetime mental and physical health issues among transgender ADSM and veterans. Data indicate that transgender ADSM report fewer lifetime mental and physical health problems than transgender veterans. Taken together, our findings suggest that more research, specifically among transgender ADSM, is needed to challenge the exclusion of transgender persons from U.S. military service based on the presumption of poor mental or physical health.

## Introduction

On June 8, 2015, the American Medical Association (AMA) adopted a formal policy stating that there was no medical rationale for excluding transgender individuals from openly serving in the U.S. military.^1^ In so doing, the AMA joined scholars, advocates, and military personnel questioning the medical and psychological fitness policies used to prohibit transgender persons from service.^2,3^ Although current policy restrictions exclude transgender people from enlistment and serving openly as transgender, population-level data suggest that up to 15,500 transgender people are active-duty service members (ADSM) or in the National Guard or Reserve forces.^4,5^ In addition, data from the National Transgender Discrimination Survey (NTDS) suggest that transgender people are twice more likely to serve in the military than members of the general population.^6^ In response to the growing support for open transgender service, the U.S. Defense Secretary Ashton Carter announced in an official memorandum (date effective from July 13, 2015) that the U.S. military would examine the current ban on open transgender service over the next 180 days, with the anticipation of policy reform expected in the spring of 2016.^7^

A number of scholars have questioned the justification for using medical and psychological fitness policies to exclude transgender individuals from military service.^2,3,8^ Chief among these has been the American Psychiatric Association's prior classification of transgender people as having a gender identity disorder (GID), now classified as gender dysphoria (GD), in the *Diagnostic and Statistical Manual of Mental Disorders* (DSM).^9^ Although the DSM-5 definition of GD focuses on the level of distress an individual experiences based on their discordant assigned birth sex and gender identity,^10^ the Department of Defense's current “Medical Standards for Appointment, Enlistment, or Induction in the Military Services” bar service of any person with a “Current or history of psychosexual conditions (302) including but not limited to transsexualism, exhibitionism, transvestism, voyeurism, and other paraphilia” (p. 48).^11^ As others have noted, the view that transgender people have a pathological psychosexual condition conflates transgender identity with mental illness and distress^2,3,12^ and assumes that all transgender people experience GD, which is not the case.^2,12–14^

To date, few health studies have been conducted with transgender ADSM. In part, this reflects the difficulties in collecting data under the current ban, as was the case with studying lesbian, gay, and bisexual (LGB) health under “Don't Ask, Don't Tell” (DADT), the military policy that excluded LGB persons from open service until 2011. However, a recent qualitative study with 14 transgender ADSM found that having to conceal one's gender identity itself was a significant source of distress.^14^ These findings are consistent with previous research on LGB veterans who served under DADT, which found that concealment was positively associated with experiences of depression and post-traumatic stress disorder (PTSD).^15^

Currently, the health needs of both transgender ADSM and veterans are poorly understood.^16–18^ Emerging research indicates that transgender veterans experience poorer health than the general veteran population^18,19^ and encounter discrimination in the Veterans Health Administration (VHA).^20,21^ For example, Blosnich et al. examined VHA records from 2000 to 2011 and found that suicide risk among veterans diagnosed with GID was 20 times higher than the general veteran population.^19^ To date, we know of no studies specifically examining the mental and physical health of transgender ADSM, or how their health compares to transgender veterans.

Investigating the health of transgender ADSM is a critical step in evaluating long-standing exclusion policies based on medical and psychological fitness. Although transgender veteran health data have often been used to extrapolate to ADSM,^4^ the degree to which this group's health may be generalized to transgender ADSM is unknown. Furthermore, data on both groups are necessary to develop appropriate standards of care and subsequent health and mental health interventions. The past 4 years have seen marked changes in the VHA directive governing transgender veteran care.^22^ Although neither sex reassignment nor reconstructive surgeries are covered, the directive guarantees access to “hormonal therapy, mental healthcare, preoperative evaluation, and medically necessary postoperative care.”^22^ Notably, receiving a GD diagnosis is not required to receive care that reflects one's self-identified gender.

The present study seeks to begin address existing research gaps by examining the mental and physical health, and military histories and experiences, of a sample of transgender ADSM and veterans. Because transgender veterans can access preoperative evaluations, hormone therapy, and postoperative care, we also document the base rates and intentions to undergo transition-related health among both groups. Also, transgender outness and family support are explored for ADSM to better understand how concealment and support are spread across ADSM military and family networks.

## Methods

Transgender ADSM were recruited through private e-mail listservs hosted by Service members, Partners, Allies for Respect and Tolerance for All (SPARTA) and snowball sampling among the SPARTA members (>4000 members). Transgender veterans were recruited using in-person recruitment at the 2013 Southern Comfort Conference in Atlanta, GA, and through snowball sampling using the Transgender American Veterans Association (TAVA) website and e-mail listserv. Data were collected through an anonymous or confidential (depending on recruitment strategy) 30-min online survey from September 2013 to September 2014. All participants provided anonymous electronic informed consent and were given the option to receive a $10 gift card. Written informed consent was waived for this study to protect participant identities. All procedures were approved by the university's institutional review board.

### Measures

#### Sociodemographic variables

Participants reported their age, gender identity, sexual orientation, and sexual attraction.

#### Military history

Participants reported their military branch of service, highest rank/grade served (e.g., E-, O-), and number of years in service.

#### Lifetime diagnosed mental and physical health problems

ADSM and veterans reported lifetime diagnoses of the following: depression, anxiety, PTSD, substance abuse, psychological adjustment issues, back problems, knee problems, asthma, upper respiratory disease, endocrine disorder, chronic obstructive pulmonary disease, osteoporosis/osteopenia, neurological conditions, rheumatoid arthritis, glaucoma, cataracts, lung disease, cardiovascular disease, congenital anomalies, and cancer (0=no, 1=yes). Additional medical health items included current height and weight, calculated as body–mass index (BMI), past year hospitalizations, and current medical coverage.

#### Transition-related health

Transition-related medical health history examined current use of hormone-replacement therapy (HRT) and having undergone gender reassignment surgery (GRS). For those who indicated no transition-related healthcare, an additional question assessing future GRS intentions was asked. Response options were 0=no, 1=yes.

#### Outness and family support

ADSM described their degree of outness to immediate family, nonmilitary friends, military within-unit friends, whole military unit, commanding officer, and all other military personnel. Responses range from 0=Not at all to 4=Completely “out” and were recoded as 0=Not Out/Limited Outness, 1=Out/Open. ADSM also described their level of family support. Responses ranged from 0=Not at all supportive to 3=Very supportive and were recoded as 0=No/Low support, 1=Moderate/High Support.

### Data analysis

Descriptive statistics analyzed proportions and central tendencies for all demographic and military characteristics. Group comparisons were conducted using chi-square and Fisher's exact tests for binary categorical variables and *t*-tests and Mann–Whitney *U* tests for continuous variables. A Holm-modified Bonferroni correction was applied with *p*<0.025 criteria to the interpretation of group comparisons for mental health, physical health, BMI, hospitalizations, and transgender transition-related health to account for familywise error rates.^23^ In addition, bivariate correlations were calculated for health, transition-related health, years of service, and outness and family support. All data were analyzed in SPSS 21.0.

## Results

### Participants

Table 1 depicts demographic and military characteristics of the 106 participants. ADSM comprised 55 persons aged 20–50 years, and veterans were 51 people aged 21–71 years. Veterans were significantly older than ADSM (Table 1). The most commonly selected gender identity was transgender woman, with a significant difference in gender identity between ADSM and veterans (*χ*^2^=11.29, *p*=0.02). There were no significant differences in sexual attraction or orientation. Among both groups, the majority identified as heterosexual and only attracted to women.

**Table 1.** **Sample Characteristics for Transgender ADSM and Veterans (*N*=106)**

Demographic	Active duty (*n*=55)	Veterans (*n*=51)	*p*
Median age (mean ±)	27.0 (29.3±7.6)	43.0 (44.0±14.3)	<0.001
Gender identity, yes, % (*n*)			0.024
Transgender woman	52.8 (28)	68.9 (31)	
Transgender man	32.1 (17)	8.9 (4)	
Woman	5.7 (3)	2.2 (1)	
Man	7.5 (4)	8.9 (4)	
Gender queer	1.9 (1)	11.1 (5)	
Sexual orientation, yes, % (*n*)			0.111
Heterosexual/straight	55.1 (27)	33.3 (15)	
Lesbian/gay/homosexual	12.2 (6)	17.8 (8)	
Bisexual	18.4 (9)	37.8 (17)	
Asexual	4.1 (2)	6.7 (3)	
Queer	10.2 (5)	4.4 (2)	
Attraction, yes, % (*n*)			0.110
Women only	56.3 (27)	36.4 (16)	
Women and men	27.1 (13)	34.1 (15)	
Men only	14.6 (7)	13.6 (6)	
Transgender women only	2.1 (1)	11.4 (5)	
None of the above	0.0	4.5 (2)	
Median years of service (mean ±)	7.0 (8.5±5.9)	6.0 (8.2±6.7)	0.268
Military branch, yes, % (*n*)			0.780
Air force	14.8 (8)	8.2 (4)	
Army	50.0 (27)	53.1 (26)	
Marine	9.3 (5)	6.1 (3)	
Navy	24.1 (13)	30.6 (15)	
Multiple branches	1.9 (1)	2.0 (1)	
Highest rank/grade served, yes, % (*n*)			0.079
E-1	0.0	2.0 (1)	
E-2	0.0	3.9 (2)	
E-3	7.4 (4)	5.9 (3)	
E-4	18.5 (10)	33.3 (17)	
E-5	29.6 (16)	25.5 (13)	
E-6	13.0 (7)	7.8 (4)	
E-7	5.6 (3)	2.0 (1)	
E-8	0.0	2.0 (1)	
O-1	5.6 (3)	0.0	
O-2	7.4 (4)	2.0 (1)	
O-3	13.0 (7)	5.9 (3)	
O-4	0.0	3.9 (2)	
O-5	0.0	2.0 (1)	

ADSM, active-duty service members.

As shown in Table 1, transgender ADSM and veterans served in all branches of the military and at multiple ranks and grades. There were no significant differences for years of service, military branch, or highest rank/grade served. In both groups, the majority had served, or was currently serving, in the army, followed by the navy. In addition, the majority in each group reported E4 (e.g., Specialist or Corporal-Army; Petty Officer Third Class-Navy) or E5 (e.g., Sergeant-Army; Petty Officer Second Class-Navy) as their highest rank of service.

### Lifetime diagnosed mental and physical health problems

Table 2 presents comparison data on mental and physical health. Depression and anxiety were the two most commonly reported conditions, followed by PTSD. A greater percentage of veterans than ADSM reported depression (64.6% vs. 30.9%, *χ*^2^=11.68, *p*=0.001) and anxiety (41.3% vs. 18.2%, *χ*^2^=6.54, *p*=0.011). In addition, 15.9% of veterans versus 1.8% of ADSM (*χ*^2^=6.53, *p*=0.011) had been diagnosed with a substance abuse disorder. There were no differences in psychological adjustment, and lifetime diagnoses of PTSD were not significant after the Holm-modified Bonferroni correction.

**Table 2.** **Lifetime Diagnosed Mental and Physical Health Problems Among Transgender ADSM and Veterans (*N*=106)**

	Active duty (*n*=55)	Veterans (*n*=51)	
Health problem	Yes, % (*n*)	Yes, % (*n*)	*p*
Mental health problems			
Depression	30.9 (17)	64.6 (31)	0.001
Anxiety	18.2 (10)	41.3 (19)	0.011
PTSD	14.5 (8)	31.1 (14)	0.047^a^
Substance abuse	1.8 (1)	15.9 (7)	0.011
Psychological adjustment issues	5.5 (3)	6.8 (3)	0.778
Physical health problems			
Back problems	16.4 (9)	27.1 (13)	0.185
Knee problems	23.6 (13)	25.0 (12)	0.872
Asthma	3.6 (2)	11.4 (5)	0.136
Upper respiratory disease	1.8 (1)	9.1 (4)	0.101
Cardiovascular disease	1.8 (1)	6.5 (3)	0.227
Endocrine disorder	1.8 (1)	6.5 (3)	0.209
Chronic obstructive pulmonary disease	0.0	9.1 (4)	0.022^a^
Rheumatoid arthritis	0.0	6.5 (3)	0.055
Osteoporosis/osteopenia	0.0	4.5 (2)	0.110
Neurological conditions	0.0	4.5 (2)	0.110
Cataracts	0.0	4.5 (2)	0.114
Glaucoma	0.0	2.3 (1)	0.261
Lung disease	0.0	2.3 (1)	0.261
Congenital anomalies	0.0	2.3 (1)	0.261
Blood disorder	0.0	2.3 (1)	0.267
Cancer	0.0	2.3 (1)	0.261
Body–mass index	24.9±2.8	28.4±5.5	0.004
Hospitalized within the past year	17.4 (8)	31.1 (14)	0.126
Transition-related health			
Currently on HRT	47.8 (22)	67.6 (25)	0.107
Have undergone GRS	4.4 (2)	19.4 (7)	0.047^a^
Intend to have GRS	53.7 (22)	35.7 (10)	0.141

^a^ Not significant with Holm–Bonferroni correction.

GRS, gender reassignment surgery; HRT, hormone-replacement therapy; PTSD, post-traumatic stress disorder.

Analyses of physical health revealed few differences. Back and knee problems were the two most commonly cited health problems. The only areas where significant differences emerged were in BMI. Here, veterans reported a higher BMI [mean (*M*)=28.4] than did ADSM (*M*=24.9) (*t*=−3.84, *p*<0.001). There were no significant differences in reported hospitalization in the past year. The majority of veterans (59.1%) received a lifetime diagnosis of a health problem under current veteran medical coverage. For ADSM, 47.7% of lifetime diagnoses were made using U.S. military medical coverage (i.e., TriCare).

Linear regression was conducted to account for age differences when examining the association between military status (ADSM/veteran) and lifetime mental and physical health problems. Models suggested that military status was marginally associated with lifetime mental health problems (*β*=0.197, *p*=0.086), but not with lifetime physical health problems (*β*=0.176, *p*=0.861).

### Transgender transition-related health

Although 47.8% of ADSM were currently on HRT, only 4.4% had undergone GRS. However, 53.7% of ADSM intended to have GRS in the future. For transgender veterans, 67.6% were currently on HRT. Still, only 19.4% had undergone GRS and 35.7% intended to have GRS in the future. There were no significant differences for HRT and GRS intentions. Although more veterans had undergone GRS than ADSM, the difference was not significant after the Holm-modified Bonferroni correction (*p*=0.047).

### Transgender outness and family support

Transgender outness was assessed only among ADSM as an indicator of identity concealment under the current ban on open service (Table 3). A majority (72.2%) of ADSM reported being out to immediate family members and nonmilitary friends (69.4%). However, only 16.2% were out to military within-unit friends. Even fewer reported being out to their whole military unit (8.1%), commanding officer (8.1%), and all other military personnel (5.6%). Despite high levels of family outness, only 37.8% of ADSM reported having moderate to strong levels of family support.

**Table 3.** **Transgender “Outness” Among ADSM (*n*=55)**

Transgender “outness” to …	Yes “Out”, % (*n*)
Immediate family	72.2 (26)
Nonmilitary friends	69.4 (25)
Military within unit friends	16.2 (6)
Whole military unit	8.1 (3)
Commanding officer	8.1 (3)
All other military personnel	5.6 (2)
Family support	37.8 (14)

### Bivariate correlations

Table 4 depicts bivariate correlations among demographic characteristics, mental and physical health problems, transition-related care, and outness and family support for ADSM. Correlations revealed that age, years of military service, mental health problems, and physical health problems were all positively correlated (*p*<0.05). More specifically, age was correlated with having been diagnosed with any mental health (*r*=0.33, *p*=0.02) and physical health (*r*=0.46, *p*=0.001) problems. Having been diagnosed with any mental health problem also was correlated with diagnoses of physical health problems (*r*=0.55, *p*<0.001), alluding to co-occurring health problems. For transition-related care, current HRT use was negatively correlated with being out to the whole military unit (*r*=−0.34, *p*=0.04) and one's commanding officer (*r*=−0.34, *p*=0.04). Being out to military within-unit friends was correlated with being out to the whole military unit (*r*=0.67, *p*<0.001), commanding officer (*r*=0.67, *p*<0.001), and all other military personnel (*r*=0.54, *p*=0.001). Being out about transgender identity was correlated with having family support (*r*=0.64, *p*<0.001).

**Table 4.** **Bivariate Correlations Between Health Problems, Transition-Related HRT, Transgender “Outness,” and Family Support Among Transgender ADSM (*n*=55)**

Items	1	2	3	4	5	6	7	8	9	10	11	12
1. Age	—	0.84^a^	0.33^b^	0.46^a^	−0.22	0.14	0.18	−0.11	−0.05	0.00	−0.05	0.26
2. Years of military service		—	0.34^b^	0.55^a^	−0.28	0.04	0.26	−0.03	0.08	0.05	0.08	0.03
3. Mental health problem			—	0.37^a^	0.20	0.12	0.14	0.13	−0.02	−0.02	0.08	−0.12
4. Physical health problems				—	−0.09	0.01	−0.12	−0.24	−0.16	−0.16	−0.13	0.03
5. Currently on HRT					—	−0.15	−0.25	−0.20	−0.34^b^	−0.34^b^	−0.27	−0.31
6. Immediate family						—	0.17	0.27	0.18	0.18	0.14	0.64^a^
7. Nonmilitary friends							—	0.31	0.21	0.21	0.17	0.12
8. Military within unit friends								—	0.67^a^	0.67^a^	0.54^a^	0.11
9. Whole military unit									—	0.64^a^	0.80^a^	0.19
10. Commanding officer										—	0.36^b^	0.18
11. All other military personnel											—	0.15
12. Family support												—

^a^ *p*<0.01.

^b^ *p*<0.05.

For transgender veterans, age was correlated with number of years of military service (*r*=0.31, *p*=0.03) and having been diagnosed with any physical health problems (*r*=0.33, *p*=0.04). As with ADSM, diagnoses of mental and physical health problems were correlated (*r*=0.54, *p*<0.001). Moreover, currently being on HRT was correlated with having undergone GRS (*r*=0.35, *p*=0.03).

## Discussion

This study is one of the first to examine health differences between transgender ADSM and veterans. Findings revealed that transgender ADSM and veterans represent the breadth of military service and branches, a full spectrum of military ranks, and a wide range of years of military service. Overall, ADSM indicated few mental and physical health problems compared to veterans. For both ADSM and veterans, physical health was positively correlated with age and years of service, indicating that as service members age and/or continue to serve, the propensity for physical health problems may increase. It is unknown if these problems reflect length of service and concomitant strain under the current ban, declines associated with length of service/deployment and/or age, or length of time exposed to minority stressors. In general, our findings are consistent with the larger body of data on the association between length of service and poor health,^24,25^ and point to the need to provide transgender ADSM and veterans with health and mental health services that meet their complex needs.

Overall, the ADSM and veterans in our sample were relatively physically healthy. The two most commonly cited physical health conditions, back and knee problems (e.g., musculoskeletal conditions), are a leading cause of medical visits, hospitalizations, and lost duty days among the broader military populations.^26,27^ In the present study, the four most commonly cited mental health problems, depression, anxiety, PTSD, and substance use, also parallel the mental health of nontransgender ADSM and veterans.^28–31^ Taken together, these results do not support the notion of poor mental or physical health as primary grounds for excluding or discharging transgender people from active-duty service.

Data from nonmilitary transgender studies have demonstrated a relationship between identity concealment and poor health.^32^ Although a large proportion of ADSM were out to family and nonmilitary friends, few were out to military friends, commanding officer, or other personnel. At the same time, our data suggest that some transgender ADSM are embedded in units where their gender identity is known, as there were high correlations between outness to within-unit friends and one's whole military unit. Although outness was significantly correlated with being out to one's commander, the magnitude was much lower and outness in the military was not correlated with family outness. This pattern likely reflects the current ban on open transgender service. In addition, the higher proportion of ADSM out to family and friends points to a high burden of identity concealment across ADSM different social networks, which has been associated with poor mental and physical health in other sexual and gender minority populations.^33^

Our findings are consistent with qualitative research with transgender ADSM, which has found that transgender personnel are out and find acceptance among some units and commanding officers.^14^ This pattern also has been identified in research with LGB ADSM and veterans, where studies indicated that many LGB personnel were out and accepted in their units and that disclosure of sexual orientation is associated with greater unit cohesion, morale, and task completion.^34–36^ For example, a recent study indicated that the DADT repeal appears to have had an overall positive impact on the military.^34^

### Limitations

Although this study provides initial insights on the health of transgender ADSM and veterans, it is important to note its limitations. First, participants comprised a convenience sample self-reporting cross-sectional data; thus, causal inferences cannot be made and findings may not generalize to the entire transgender ADSM and veteran population. The present study did not assess assigned birth sex, but rather relied on self-identification. Given the small sample size, we are unable to draw meaningful comparisons among subsets of self-identified transgender men or women ADSM. We also did not inquire the number of deployments among ADSM and veterans, which has been associated with mental and physical health problems.^37^ Although length of service may provide insights into deteriorating health over time, the number and length of deployments and dwell time between deployments are important factors to consider in future research.^24,25^ In addition, our study relied on a self-reported checklist of lifetime health diagnoses, rather than inventories or validated measures of existing health conditions. Furthermore, our questions regarding transgender transition-related care were limited to the physical aspects of transgender transition (e.g., HRT, GRS). Additional research is needed to better understand the use of transition-related counseling and therapy among transgender ADSM and veterans.

We also did not examine how specific demographic factors, such as race/ethnicity, income, and education, were related to health. These factors were intentionally omitted from the survey to limit identifying information and offer increased confidentiality for study participants, particularly for transgender ADSM. However, we recognize that these data are important to obtain, as prior work has observed ethnoracial disparities among transgender veterans.^38^ As with other primary data on transgender samples, this study had a relatively small sample size compared to larger secondary analyses of transgender veterans.^4,18–20^ Thus, the current sample is not representative of all transgender ADSM and veterans, as no representative sample of either group currently exists. Whereas ADSM are included in the NTDS, reports of mental and physical health diagnoses that serve as the basis for subsequent research on health needs and disparities are not included.^19^

Recruiting transgender ADSM remains a challenge under the current ban. Similar to the restrictions imposed by DADT, researchers must use methods that minimize participant harm (e.g., discharge).^39^ Thus, our study represents one of the first to collect primary data with this hard to reach and vulnerable population, subsequent studies including the investigation conducted by the U.S. Department of Defense Transgender Service Member Working Group.^7^

## Conclusion

An estimated 18 countries currently allow transgender men and women to openly serve in the military.^2,40^ In March 2015, Master Chief Petty Officer of the Navy (AW/NAC) Mike Stevens indicated that transgender people should be allowed to serve “if they meet the Navy's standards.”^41^ More recently, Defense Secretary Ashton Carter recently announced that the military anticipates lifting the ban on transgender service at the conclusion and recommendation of the Secretary of Defense for Personnel and Readiness working group investigation.^42^ Holding in abeyance the conflation of transgender identity with a psychosexual condition that requires psychiatric and/or psychological treatment, our data do not indicate that transgender ADSM report levels of lifetime mental or physical health problems at rates that should necessarily bar them from service. However, additional research specifically focused on larger samples of transgender ADSM is needed to challenge the exclusion of transgender persons from serving in the U.S. military based on the presumption of poor mental or physical health. Moreover, our data suggest that transgender ADSM diverge from veterans on several important health indicators. Although additional research is needed, our study is one of the first to collect primary data on a sample of transgender ADSM and lends support to an emerging consensus that transgender men and women should be allowed to serve openly in the U.S. military with their cisgender heterosexual and LGB peers.^2,4,6,13,20,43^

## Abbreviations Used

ADSM: active-duty service members
AMA: American Medical Association
BMI: body–mass index
DADT: “Don't Ask, Don't Tell”
DSM: Diagnostic and Statistical Manual of Mental Disorders
GD: gender dysphoria
GID: gender identity disorder
GRS: gender reassignment surgery
HRT: hormone-replacement therapy
LGB: lesbian, gay, and bisexual
NTDS: National Transgender Discrimination Survey
PTSD: post-traumatic stress disorder
SPARTA: Service members, Partners, Allies for Respect and Tolerance for All
VHA: Veterans Health Administration
